# Supplementary material for: Genetic comparisons of interleukin-17 reveal a framework for complex signaling evolution
Source: bioRxiv. 2026 Apr 14:2026.04.13.718218. Preprint. [Version 1] doi: 10.64898/2026.04.13.718218 (PMC13105070; doi:10.64898/2026.04.13.718218)
Supplement: Supplement 3 [file media-3.zip › Supplemental files for paper/ERC/IL17xIL17_comparisons/IL17RB_IL17REL.pdf]

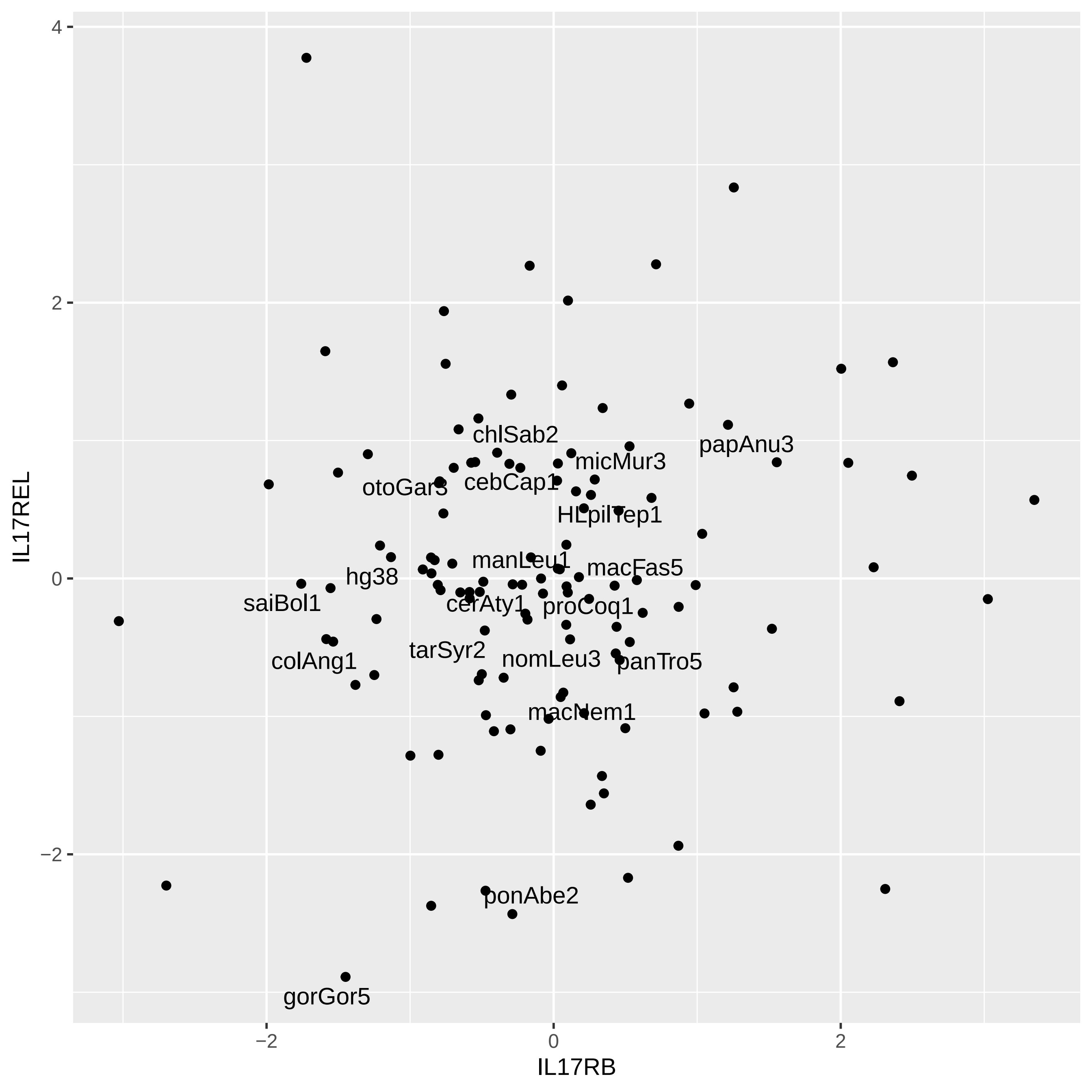

IL17REL
IL17RB
4
2
0
−2
−2
0
2
chlSab2
papAnu3
micMur3
otoGar3
cebCap1
HLpilTep1
manLeu1
macFas5
hg38
saiBol1
cerAty1
proCoq1
colAng1
tarSyr2
nomLeu3
panTro5
macNem1
ponAbe2
gorGor5
